# Plant long non-coding RNAs: identification and analysis to unveil their physiological functions

**DOI:** 10.3389/fpls.2023.1275399

**Published:** 2023-10-26

**Authors:** Edmundo Domínguez-Rosas, Miguel Ángel Hernández-Oñate, Selene-Lizbeth Fernandez-Valverde, Martín Ernesto Tiznado-Hernández

**Affiliations:** ^1^ Coordinación de Tecnología de Alimentos de Origen Vegeta, Centro de Investigación en Alimentación y Desarrollo, Hermosillo, Sonora, Mexico; ^2^ CONAHCYT, Centro de Investigación en Alimentación y Desarrollo, Hermosillo, Sonora, Mexico; ^3^ School of Biotechnology and Biomolecular Sciences and the RNA Institute, The University of New South Wales, Sydney, NSW, Australia

**Keywords:** bioinformatics, plant long non-coding RNA, characteristics of lncRNA, regulatory functions of lncRNA, physiological functions of lncRNA

## Abstract

Eukaryotic genomes encode thousands of RNA molecules; however, only a minimal fraction is translated into proteins. Among the non-coding elements, long non-coding RNAs (lncRNAs) play important roles in diverse biological processes. LncRNAs are associated mainly with the regulation of the expression of the genome; nonetheless, their study has just scratched the surface. This is somewhat due to the lack of widespread conservation at the sequence level, in addition to their relatively low and highly tissue-specific expression patterns, which makes their exploration challenging, especially in plant genomes where only a few of these molecules have been described completely. Recently published high-quality genomes of crop plants, along with new computational tools, are considered promising resources for studying these molecules in plants. This review briefly summarizes the characteristics of plant lncRNAs, their presence and conservation, the different protocols to find these elements, and the limitations of these protocols. Likewise, it describes their roles in different plant physiological phenomena. We believe that the study of lncRNAs can help to design strategies to reduce the negative effect of biotic and abiotic stresses on the yield of crop plants and, in the future, help create fruits and vegetables with improved nutritional content, higher amounts of compounds with positive effects on human health, better organoleptic characteristics, and fruits with a longer postharvest shelf life.

## 1 Introduction

With the advance in the study of fungi, plants, and animal genomes, it was noted that a large proportion of their genomes is transcribed, yet a great number of the RNA transcripts showed a null capacity to code for proteins. These transcripts are known in general as non-coding RNAs (ncRNAs). Among these ncRNAs, there are the long non-coding RNAs (lncRNAs), which are primarily defined as having a size of 200 nt or more and a null capacity to code for proteins (Morris and Mattick, 2014; Chekanova, 2015). Even though lncRNAs are prevalent across eukaryotes (Kapusta and Feschotte, 2014; Morris and Mattick, 2014), for the vast majority of them, experimental evidence of their different functions has just been generated in recent years (Cabili et al., 2011; Pauli et al., 2011).

In general, the study of lncRNAs is challenging due to their low expression and little conservation at the sequence level, in comparison with messenger RNA (Kapusta and Feschotte, 2014; Chekanova, 2015). However, with the recent advances in RNA-seq and bioinformatics technologies, our capacity to study these elements and to elucidate their importance in the regulation of gene expression in different organisms has been greatly improved (Signal et al., 2016). Historically, lncRNAs were first described in animal models and found to possess a regulatory function such as control at transcription and post-transcriptional levels (Wang et al., 2008; Pauli et al., 2011). In general, lncRNAs have been more studied on animal models (humans, mice, etc.) and less on plants (Zhao et al., 2021; Li et al., 2022). In the case of plants, lncRNAs have been reported on Arabidopsis and crop plants such as cotton, wheat, rice, and maize (Chekanova, 2015; Bai et al., 2019; Yang G. et al., 2022; Yang X. et al., 2022; Li et al., 2022; Zhang et al., 2022). In plants, lncRNAs are transcribed mainly by the RNA polymerases I, II, and III. Although polymerase II principally transcribes mRNA, it can also transcribe lncRNAs. Furthermore, plants also have the polymerases IV and V, which give rise to lncRNA, which appears to function in transposable element silencing (Wierzbicki et al., 2021). Similar to protein-coding transcripts, lncRNAs possess splicing signals and promoter regions, and their mature form may have a 5´-cap and a polyadenylated tail on their 3´ end (Morris and Mattick, 2014). Altogether, the same machinery and signaling involved in the synthesis of protein-coding transcripts is shared by lncRNA transcripts (Wierzbicki et al., 2021). Moreover, these plant-specific lncRNAs are not entirely understood, but they were found to be essential for carrying out RNA-directed DNA methylation, a crucial adaptation mechanism in plants (Matzke and Mosher, 2014; Zhou and Law, 2015). Furthermore, transcripts generated by the Pol V in Arabidopsis induce the formation of heterochromatin-forming complexes through sequence complementarity to carry out silencing of nearby genes. These lncRNAs lack the polyadenylated tail on their 3´ end and they can be tri-phosphorylated or have capped 5´ends (Wierzbicki et al., 2008). Even if the function of lncRNAs as regulators of diverse mechanisms in plants has been established (Zhang X. et al., 2019), the conservation and evolution of these genetic elements has been hard to study due to the lack of sequence homology (Derrien et al., 2012; Mattick and Rinn, 2015; Kashi et al., 2016). However, lncRNAs not conserved by sequence can arise from the same genomic region and, thus, be conserved by position (i.e., syntenic) (Mohammadin et al., 2015; Deng et al., 2018; Palos et al., 2022). Additionally, many lncRNA functions are centered on the capacity to fold into secondary structures, which allows them to interact with other types of RNA, DNA, and proteins (Qian et al., 2019).

Thanks to the advances in genomics, transcriptomics, and bioinformatics, a great number of lncRNAs have been identified and associated with biological functions in plants. The search for orthology on lncRNA transcripts from different species has just begun to be explored and remains a challenge due to the features of these non-coding elements of the genome (Cabili et al., 2011; Budak et al., 2020). LncRNAs are less abundant than other RNAs element (rRNA, mRNAs, etc.) and are highly tissue-specific and even cell-specific (Flynn and Chang, 2014). Therefore, the lncRNA study was only possible due to the development of next-generation sequencing technologies such as Illumina, PacBio, Ion Torrent, and Nanopore (Kang and Liu, 2015; Wang et al., 2015a; Deshpande et al., 2019). These technologies not only enabled the sequencing of lncRNAs but also expedited the completion of plant genomes, which, to date, account for more than 600 (Sun et al., 2021), making the genome-wide study of lncRNAs a feasible prospect. Whole genomes are essential to identify syntenic regions of similar evolutionary origin, which can then be queried for producing lncRNAs that might be orthologues but may have substantially diverged in sequence while retaining the same function (Quinn et al., 2016; Ulitsky, 2016; Ramírez-Colmenero et al., 2020).

Several plant lncRNAs have been shown to be involved in the regulation of the whole genome gene expression (Danjing et al., 2022).

Below, we describe some characteristics of the lncRNA, the bioinformatics tools developed to study them, and several examples of the physiological role of plant lncRNAs.

## 2 Classification of lncRNAs

**Figure 1:**
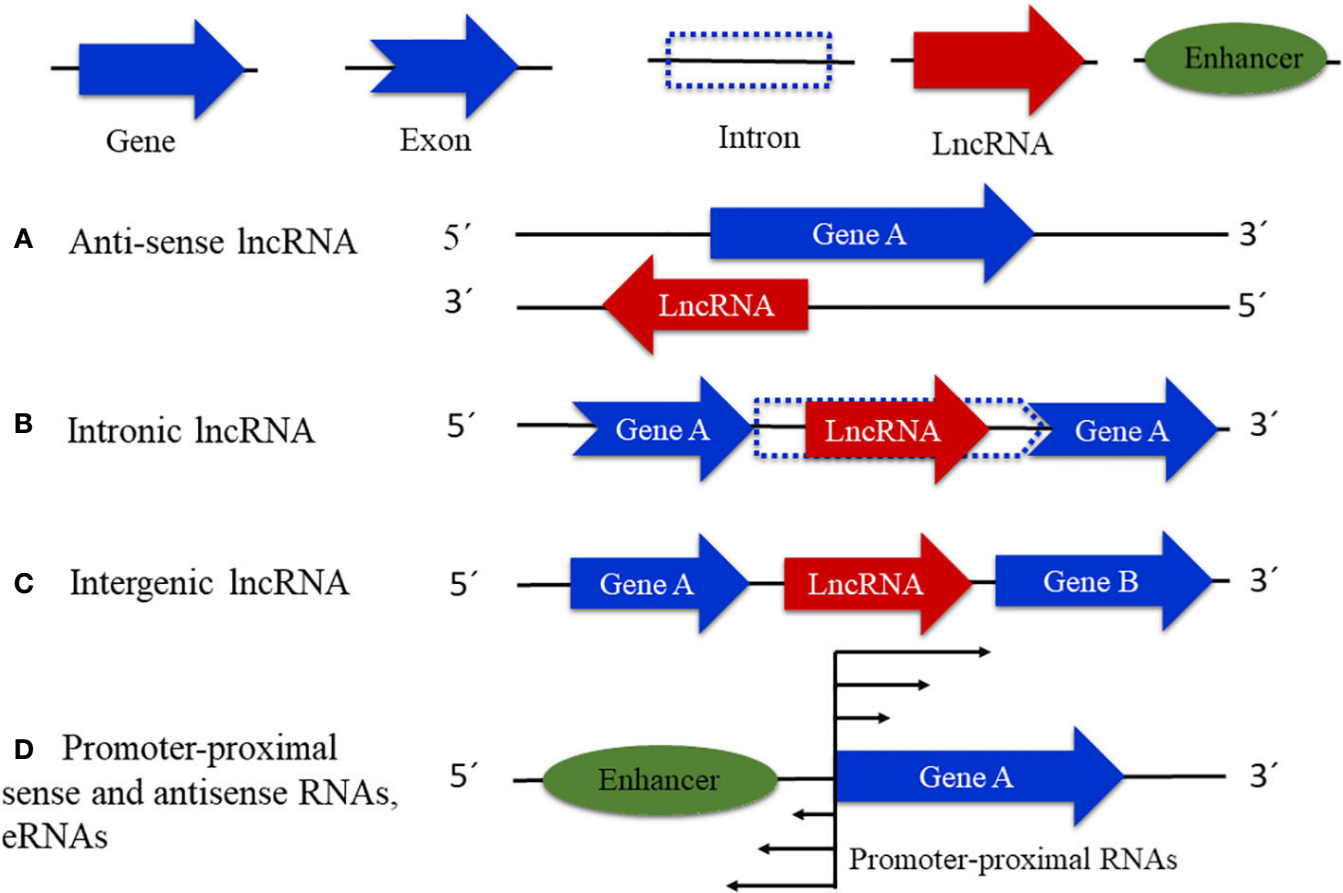
Types of known lncRNAs. **(A)** Natural antisense transcripts that are associated with the antisense strand of a protein-coding gene. **(B)** Intronic lncRNAs that are transcribed from intronic regions. **(C)** Intergenic lncRNAs, associated with intergenic DNA regions. **(D)** Enhancer RNAs (eRNAs) and promoter-proximal noncoding RNAs in either sense or antisense.

With the discovery of a great number of ncRNA molecules, different from those with housekeeping function like ribosomal RNA, transfer RNA, and small nuclear RNA (Morris and Mattick, 2014), the problem of labeling these new regulatory elements, including lncRNAs, emerged. LncRNAs can be classified based on the position of lncRNA transcripts in relation to adjacent protein genes (Mattick and Rinn, 2015) into natural antisense transcripts (NATs), which are associated with the antisense strand of protein-coding DNA, intronic (incRNAs), and intergenic (lincRNAs), which are encoded by introns and intergenic regions, respectively (Mattick and Rinn, 2015; Deng et al., 2018) (**Figure 1**). Other classifications of lncRNAs include those present near transcription start sites (TSSs), transcription termination sites (TTSs), and close or overlapping with enhancer regions (eRNAs). Furthermore, it is important to mention that this classification is rudimentary and leaves out many other possible types of lncRNAs (Kashi et al., 2016), for example, the lncRNAs encoded by transposon regions that have been described in Arabidopsis, rice, and maize (Fort et al., 2021); lncRNAs that operate as precursors or targets of small interfering RNAs (siRNAs) (Zhang P. et al., 2019; Zhang and Zhu, 2014); and others that are yet to be described.

In plants such as *Arabidopsis thaliana*, *Arabidopsis lyrata*, *Populus trichocarpa*, and *Zea mays*, approximately 80% of lncRNAs so far studied fall in the category of lincRNAs and only approximately 20% have been classified as incRNAs or NATs (Bai et al., 2019; Li C, et al., 2023). Furthermore, out of these NATs, approximately 70% are encoded by DNA regions that overlap with protein-coding genes almost entirely or with complementary sequences in their 5´ or 3´ ends as shown in studies carried out in Arabidopsis, buckthorn, maize, and other species (Li et al., 2014; Wang et al., 2014a; Zhang G. et al., 2018). However, even if the identification of the distinct types of lncRNAs in plants has been reported, the number of transcripts described is probably underestimated, due to the lack of the complete genome sequence of most plants (Kashi et al., 2016).

## 3 Approaches for the identification of lncRNAs

The study of plant lncRNAs is growing and is primarily focused on model organisms and crops of economic interest (Patra et al., 2022). The identification of lncRNAs is based on filtering out RNA transcripts that exhibit characteristics present in mRNAs and other types of ncRNAs (rRNAs and snRNA, among others), removing those with identifiable protein domains and/or large open reading frames, and annotating the remaining transcripts as potential lncRNAs (Chekanova, 2015). This is done through the implementation of an array of different sequencing technologies and bioinformatic tools. However, given the complexity of these molecules, this approach can fail to identify lncRNAs with special features, for instance, lncRNAs with long open reading frames that are not translated, or the capacity to code for small peptides (Cabili et al., 2011; Tripathi et al., 2017; Zhang P, et al., 2019). Other problems in the identification of lncRNAs can be the misidentification of the molecules as coding genes. For example, recently, it was noted that some lncRNAs were erroneously classified as protein-coding transcripts on the database Araport11, a widely used database of *A thaliana* (Cheng et al., 2017; Corona-Gomez et al., 2022). Among the misidentified lncRNAs, there is the lncRNA *IPS1 (INDUCED BY PHOSPHATE STARVATION 1)*, which is a well-characterized lncRNA involved in phosphate homeostasis, and the lncRNA *APOLO* (*AUXIN-REGULATED PROMOTER LOOP*), which has also been experimentally characterized and is involved in lateral root formation in response to auxin (Zhang Z. et al., 2019; Ariel et al., 2020; Corona-Gomez et al., 2020).

Based on the above, researchers must be careful in the design of pipelines for the identification of these elements, particularly when derived from next-generation sequencing data. Plant lncRNAs are often related to the regulation of development stages or in the response to different environmental stress. Therefore, it is important to include many different transcriptomes in the sequencing (Liu et al., 2015; Wierzbicki et al., 2021). This wide range of action creates the necessity of sequencing different tissues and stages of development to improve and increase the sensitivity of lncRNA identification. Also, another point to keep in mind is that lncRNAs interact very strongly with other molecules (RNA-DNA-proteins). Thus, approaches with few tissues or time points can be insufficient to characterize the elements fully. This highlights the necessity of using hybrid sequencing methods, including short- and long-read sequencing technologies (König et al., 2010; Rodrigues et al., 2020; Smolka et al., 2021) to get a complete characterization.

## 4 Bioinformatic identification of lncRNAs

The basic pipeline for the identification of lncRNAs starts with the evaluation of the raw reads from RNA-Seq experiments, which can be aligned to a reference genome using software such as Bowtie, in case the reference genome is available (Kashi et al., 2016). If no reference genome is available, the *de novo* assembly of transcriptome can be used as an alternative; this method is less accurate due to errors in the sequencing, especially the creation of chimeric transcripts, which may be an impediment to the correct identification of lncRNAs because lncRNAs tend to be close to mRNA transcripts (Kashi et al., 2016; Deshpande et al., 2019).

Assembled transcripts are then filtered out with different software that takes into account the characteristics of lncRNAs to differentiate between protein-coding gene and putative lncRNAs (Cabili et al., 2011; Zhang G. et al., 2018). The identification of lncRNA starts with the filtering of transcripts lesser than 200 nt, while the rest of the transcripts are compared to known protein-coding genes with tools like BLAST to eliminate all transcripts that show homology with known coding genes (Camacho et al., 2009). Furthermore, other filters are utilized to analyze the remaining transcripts like the elimination of those transcripts with an open reading frame (ORF) greater than 100 amino acids (Li et al., 2020).

**Figure 2:**
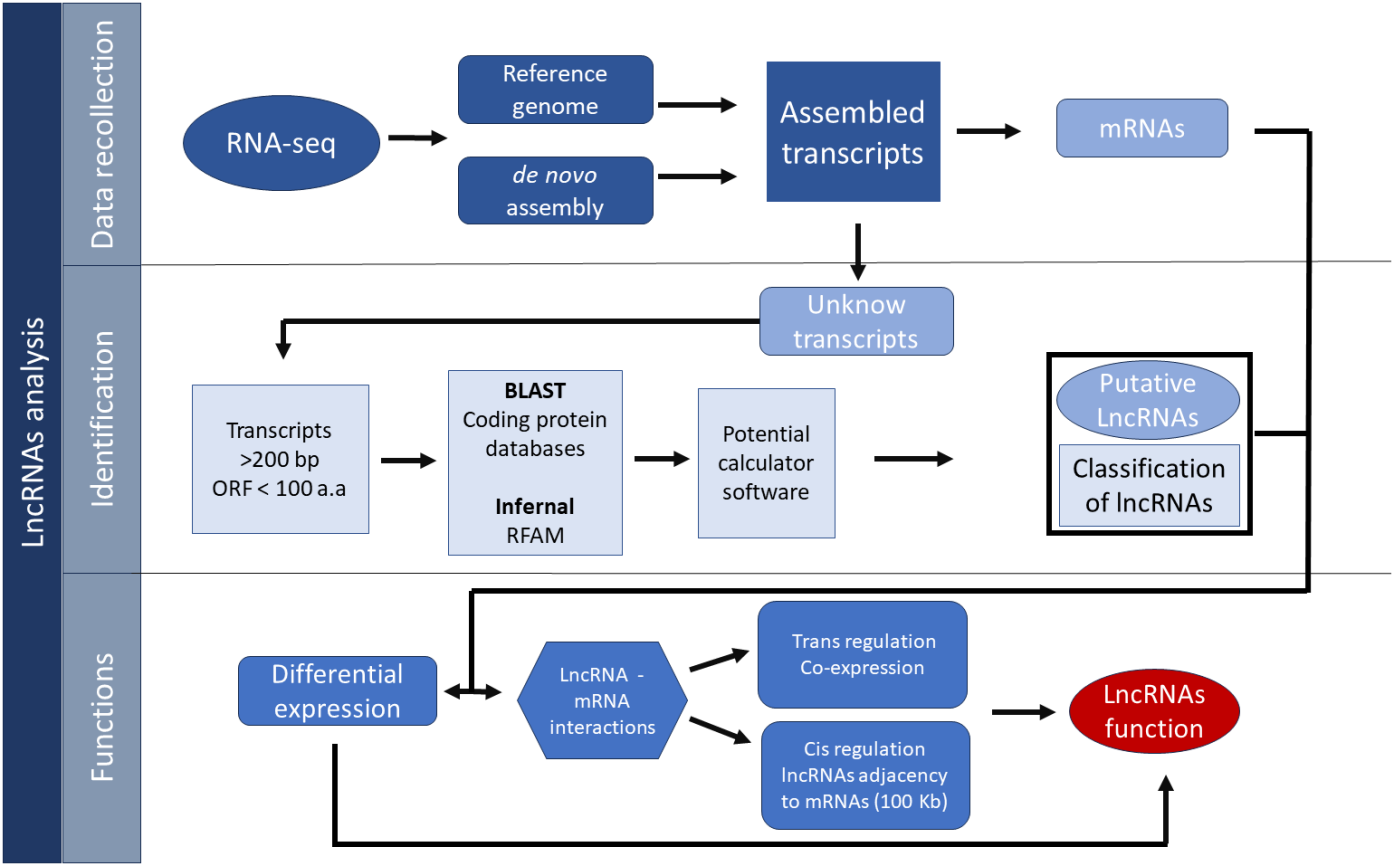
General pipeline for the identification of lncRNAs.

Transcripts that achieve the ORFs’ size criteria can also be assessed for protein-coding potential, by using the software coding potential calculator (CPC). This software uses a machine-learning approach that was trained with the database UniProt Reference Clusters (UniRef90) and ORF features like codon bias, integrity, and coverage, which give a score that indicates the possible coding capacity of a given transcript (Kashi et al., 2016; Lin X. et al., 2019). Other software that evaluates the coding capacity that can be used in conjunction with CPC utilizes distinct features for the same goal, such as the CPC2 software, which is an upgraded version of CPC that considers databases of numerous species from plants among other organisms to diminish the bias toward animal genes of the software CPC. Other additional features of CPC2 are the Fickett score, length, integrity, and isoelectric point of the ORFs in the transcripts (Kang et al., 2017). Additional software tools include the coding-potential assessment tool (CPAT), which uses a logistic regression model that integrates four features such as ORF length, coverage, Fickett score, and Hexamer usage bias (Wang et al., 2013). Owing to the complexity of these non-coding molecules, a common pipeline usually includes the use of two or more of the tools mentioned (Wang et al., 2015a) to increase the astringency and get a more accurate outcome (**Figure 2**).

In addition to the above mentioned, other non-coding transcripts like tRNAs can be filtered out with tools like INFERNAL, a software that uses covariance models (CMs) for input sequences to look for homology bases in the secondary structure in databases like the ncRNA family database known as RFAM (Nawrocki and Eddy, 2013; Kalvari et al., 2018; Kalvari et al., 2020; Kalvari et al., 2020). Lastly, once the lncRNAs have been classified, some researchers filter out exonic lncRNAs because the overlapping of exons with coding genes can lead to a false-positive identification of lncRNAs (Kashi et al., 2016).

Once an lncRNA or a set of them has been identified with bioinformatic tools, further experiments can be done to study their function on a deeper level. Among this, the most common approaches are gain and loss of function experiments. Gain of function might involve the overexpression of lncRNA. In this context, the overexpression of the lncRNA *lncY1* in *Betula platyphylla* showed that it induces salt tolerance by binding to the promoters of the transcription factors *BpMYB96* and *BpCDF3* (Jia et al., 2023). Loss of function might be queried by eliminating the lncRNA expression using CRISPR. Indeed, using this approach, it was possible to eliminate 1,325 bp of the *lncCOBRA1* and demonstrate its function in germination (Kramer et al., 2022) or by the study of T-DNA insertion mutant. For instance, by studying a rice T-DNA mutant, it was possible to demonstrate that the alteration in seed development was due to the loss of function of *MISSEN* lncRNA (Zhou et al., 2021). As it was just mentioned, both of these approaches are followed up by phenotypic characterization and validation experiments. Other techniques, such as fluorescence *in situ* hybridization (FISH), may be used to visualize the cellular localization of the RNA, which, in combination with other techniques, can suggest or refine the knowledge into their molecular function.

To study lncRNAs using RNA-Seq, the experiments must be carefully designed. This includes selecting the right tissue and tissue amount to enable high-quality RNA isolation (Yockteng et al., 2013). The isolation of tissue can be achieved with micromanipulation, laser microdissection (LCM), fluorescence-activated cell sorting (FACS), and microfluid technics (Potter, 2018). An experimental alternative to the RNA-seq is the sequencing of RNA molecules from a specific cell, known as single-cell sequencing (scRNA-Seq). Other techniques that can be used in the identification and characterization of lncRNAs include the targeted sequencing of RNA molecules and their target molecules, as many lncRNAs exert their functions by interacting with other molecules in the genome and, thus, the identification of their binding partners can help in their functional characterization. Among the methods that can be used for this purpose are immunoprecipitation-based methods such as Chromatin Isolation by RNA Purification (ChIRP), where lncRNAs associated with chromatin can be sequenced (Quinn and Chang, 2015). Other experimental approaches are summarized in **Table 1**.

**Table 1:** Different experimental approaches to study the interaction of lncRNAs with different molecules.

Interaction Type	Description	References
RNA–protein		
PIP-Seq (Protein Interaction Profile Sequencing)	Based on the identification of protein-protected sites (PPSs), which reveal the interaction of proteins with RNA, and the comparison of the ratio of read coverages in double-stranded RNA (dsRNA)-seq compared to single-stranded RNA (ssRNA)-seq libraries, which can be used to infer lncRNA secondary structure.	(Shan et al., 2019)
TRAP-Seq (Translating Ribosome Affinity Purification)	Based on the sequencing of tagged ribosomes ligated to RNAs (lncRNAs); this allows the identification of lncRNAs and their polysome complex.	(Rodrigues et al., 2020; Traubenik et al., 2020)
RIP-Seq (RNA Immunoprecipitation)	Consist in the immunoprecipitation of tagged proteins and then the associated lncRNAs are isolated via basic sequencing.	(Bazin et al., 2018)
CLIP-Seq (Cross-linking Immunoprecipitation)	Work like RIP-Seq but with the implementation of UV cross-linking, which allows more resolution and mapping binding sites.	(Hafner et al., 2021; Karagkouni et al., 2021)
ChIRP-MS (ChIRP followed by mass spectrometry)	LncRNA-associated chromatin is purified using hybridized biotinylated antisense oligonucleotides against a particular lncRNA and streptavidin-coated beads. Associated proteins are identified through mass spectrometry	(Xu et al., 2021)
RNA–DNA		
ssDRIP-Seq (single-stranded DNA–RNA Hybrid Immunoprecipitation)	Immunoprecipitation of single-stranded DNA and a DNA: RNA hybrid (R-loops)	(Xu et al., 2017)
RIDP (RNA Isolation by DNA Purification)	Use of the S9.6 antibody RIP-seq to the detection of R-loops on RNA : DNA hybrids.	(Smolka et al., 2021)
DRIP-RNA-Seq	Chromatin regions of interest are purified using biotinylated probes to isolate associated RNAs. Known RNAs can be detected and quantified by qPCR. Alternatively, novel RNAs could be uncovered by sequencing	(Pacheco et al., 2021)
RNA–chromatin		
ChIRP-Seq (Chromatin Isolation by RNA Purification and Sequencing)	Chromatin isolation by RNA purification ensued by sequencing	(Ariel et al., 2020; Zheng et al., 2021)
RADICL-Seq (RNA and DNA Interacting Complexes Ligated and Sequenced)	Genome-wide RNA–chromatin interactions mapping in intact nuclei	(Ramakrishnaiah et al., 2020)
Chromatin 3D		
Hi-C (Chromosome Conformation Capture coupled with High-Throughput Sequencing)	3C-derived technique aimed to uncover chromatin interactions at genome-wide scale by sequencing	(Chen et al., 2021)
ChiA-PET (Chromatin Interaction Analysis by Paired-End Tag Sequencing	HiC-derived technique, coupled with the use of immunoprecipitation, is designed to reveal chromatin interaction networks of regions associated with a defined chromatin mark or protein of interest	(Capurso et al., 2020; Yuan et al., 2022)
HiChIRP (Hi-C coupled with Chromatin Isolation by RNA Purification)	Hi-C derived technique, coupled with ChIRP for genome-wide identification of lncRNA-associated chromatin loops	(Mumbach et al., 2019)
RNA–RNA		
RNA–RNA Interactome	RNA–RNA duplexes are enzymatically transformed into RNA chimeras	(Singh et al., 2022)

## 5 LncRNA identification drawbacks

The analysis of existing ncRNA data has already yielded many novel insights into the functions of ncRNAs. Furthermore, this genetic material, once believed to be junk in the genome, has been shown to play a role in different regulatory mechanisms, especially in the case of the lncRNAs (He et al., 2018).

The two most important challenges to studying the lncRNAs are identification and functional characterization. This is due to the specific expression of these molecules during only certain stages of development and in a specific tissue, which demands more sequencing depth in a higher number of developmental stages or different treatments. Additionally, lncRNAs are very poorly conserved at the sequence level, which affects our capacity to identify them across different species. This has been well documented in animals (Ulitsky, 2016; Ramírez-Colmenero et al., 2020), and it is even more striking in plants, as they drastically rearrange their genomes in very short evolutionary periods (Wendel et al., 2016). This limited level of conservation has made it difficult to identify *bona fide* orthologues with functionally characterized lncRNAs in one particular species. Nevertheless, some efforts have been made in this area looking for conservation of lncRNA features such as splice sites (Corona-Gomez et al., 2020), synteny (Palos et al., 2022) and secondary structure (Corona-Gomez et al., 2023) with a strong focus in the Brassicaceae. These efforts have revealed substantially higher levels of conservation than what is found only using sequence similarity approaches.

Furthermore, the functional characterization of these molecules is extremely challenging because lncRNAs can interact with an array of different molecules of the genome with little sequence complementarity, structural motifs, and other molecules as intermediaries (Hahne et al., 2021), which means that the identification of their targets requires the use of diverse experimental techniques. This results in the need of expensive and time-consuming experimental characterization of each lncRNA independently. Additionally, the molecular characterization of these elements cannot be approached as that of protein-coding genes, as there is no ORF that can simply be disrupted to try to link the phenotype with a particular protein, meaning more experiments need to be undertaken and carefully designed to demonstrate that the observed phenotypic effects are due to the lncRNA and not to the disruption of DNA-encoded regulatory elements, such as promoters, enhancers, and insulators (Kopp and Mendell, 2018).

## 6 Elucidating of lncRNA physiological functions in plants

It is well known that the biological processes of plants are under the control of a complex regulatory network. For many years, coding genes have been recognized as key players in plant biology; however, in recent years, crucial roles associated with lncRNAs have been revealed (Li et al., 2023). Interestingly, despite their lack of conservation and diverse characteristics, lncRNAs tend to share molecular functions; they can act as tethers to bring protein and epigenetic modifying complexes in proximity with their sites of action, as scaffolds for other proteins and RNAs to interact with each other, and as small RNA precursors or sponges, among others (Zhao et al., 2022).

With the goal to elucidate the function of lncRNAs, distinct approaches have been utilized. One approach is to analyze the biological functions of coding genes in the vicinity of lncRNAs, under the assumption that they may be regulated in *cis*, and their functions might reflect that of the lncRNA (Wang et al., 2015a; Subburaj et al., 2018). In this approach, coding genes are searched 100 kb upstream or downstream to the genome location of the lncRNA of interest. Another feature that can be used to assign a potential function to lncRNAs is to assess their co-expression with protein-coding genes. Furthermore, the interaction between a set of lncRNA and another set of mRNAs that participates in a specific metabolic pathway may also provide insights into their biological function (Kashi et al., 2016). Such analysis can be performed with the construction of co-expression networks using tools such as Cytoscape, which allows the identification of possible level of interaction between lncRNAs and mRNAs pairs (Kohl et al., 2011). Furthermore, with these analyses, it is possible to identify targets of the lncRNA regulated in *trans*. Additionally, since lncRNAs interact at various levels with the mRNAs that they regulate, using these approaches, many lncRNAs that regulate important biological phenomena such as plant development, and abiotic and biotic stress have been identified in plants (Patra et al., 2022). Multiple works have leveraged genomics approaches to identify lncRNA sequences associated with diverse physiological functions in plants, which represents important advances to identify and elucidate the role of lncRNAs in plant biology. These advances are described in the next section of the review.

### 6.1 LncRNAs in plant development

#### 6.1.1 Vernalization

One of the most important aspects that influence plant biology is environmental conditions, since these are determinants for plant development and growth. Flowering time is a physiological aspect that is modulated by cold conditions through the vernalization process, which has been demonstrated to be finely controlled by lncRNAs (Jampala et al., 2021). In *Arabidopsis thaliana*, this process is controlled by the flowering locus C, *FLC*, which encodes for a MADS-box transcription factor that silences the genes needed to induce the change from vegetative growth to flowering (Fujiwara et al., 2010). It has been shown that the regulation of this gene is carried out by *COOLAIR*, which is an antisense lncRNA derived from the *FLC* locus (Hawkes et al., 2016). The transcription of this lncRNA starts downstream of the poly-A site of the gene *FLC* sense transcript and is upregulated by a cold environment. This lncRNA boosts the cold-induced downregulation of the *FLC* gene (Song et al., 2012). Moreover, the *FLC* locus is controlled by an intronic lncRNA that is transcribed in sense from the first intron of *FLC*: *COLDAIR*. This lncRNA physically interacts with the polycomb repressive complex 2, PCR2, composed of six proteins with the goal to target the complex to the *FLC* locus. In this way, *COOLAIR* and *COLDAIR* induce a stable and quantitative epigenetic repression (Heo and Sung, 2011; Song et al., 2012; Kim et al., 2017).

Furthermore, to control the expression of the *FLC* gene, there is another lncRNA that is transcribed upstream of the translation start site, at the promoter region, designated as *COLDWRAP*. This lncRNA interacts with the PCR2 complex by a motif located at the 5´ site. Furthermore, *COLDWRAP* along with *COOLAIR* maintains the PCR2 complex interacting with the promoter by the formation of a loop that maintains the *FLC* locus silenced (Kim and Sung, 2017). The control by lncRNA of the cold-induced gene *MAF4*, which plays a role in avoiding early vernalization response, has also been studied. In this regard, a natural antisense lncRNA designated *MAS*, for *MAF4* antisense RNA, was isolated from Arabidopsis tissues subjected to ABA, dehydration, and cold treatments. The transcription of *MAS* starts a few bases from the *MAF4* transcription terminator signal and finishes within the first intron region of the *MAF4* gene. With the goal to test whether the *MAF4* gene controls the transcription of *MAS* or the opposite, studies with *MAF4* T-DNA mutants and two *MAF4* knocked-down lines as well as two *MAS* knocked-down lines were carried out. It was found that the expression of *MAF4* gene expression was reduced in the lines in which the *MAS* gene expression was eliminated. Furthermore, the expression in response to cold stress was also almost lost. Also, the *MAF4* T-DNA mutant and the *MAS* knocked-down line showed an early flowering phenotype suggesting that *MAS* induces *MAF4* expression and this, in turn, suppresses flowering (Zhao et al., 2018).

#### 6.1.2 Root development

The root is an essential organ for the plant’s adaptation to environmental conditions; it directly senses stress conditions, so its adaptation capacity is crucial to plant survival. In recent years, the role of lncRNAs in the regulation of root development has been described. A prominent example is the lncRNA designated as *APOLO* for Auxin Regulated Promoter Loop RNA, which induces the formation of a chromatin loop that includes the promoter region of the gene *PINOID* encoding a regulatory kinase controlling the polar localization of an auxin transporter. This gene is located 5,248 bp downstream of the *APOLO* gene locus. Both *APOLO* and *PINOID* are upregulated by the presence of auxin and it had been observed that the expression of these genes decreases simultaneously after 12 h of auxin treatment. Repression of *APOLO* by RNAi also eliminates the expression of *PINOID* gene and increases the time for gravitropism response, which is a phenotype similar to the *pinoid* mutants (Ariel Federico et al., 2014). Moreover, it was found that *APOLO* activates the transcription of *RHD6*, for Root Hair Defective 6, by creating a chromatin loop including the promoter region most likely through the PRC1 and PRC2 protein complexes. Furthermore, *APOLO* also interacts with the transcription factor WRKY42 to regulate the *RHD6* gene inducing in this way the growth of root hairs under stress by cold temperatures (Moison et al., 2021).

Auxin induces lateral root development in Arabidopsis. Two NSR proteins, NSRA and NSRB, for nuclear speckle RNA-binding proteins, were found to regulate the splicing pattern of genes. Interestingly, the double mutant *nsra*/*nsrb* shows a phenotype with fewer roots with reduced length and alteration in the splicing pattern of 85 genes. Furthermore, out of the 85, 11 were found to be related with the initiation of the lateral root. A transcriptome of the double mutant under auxin treatment showed the presence of 2,200 genes with different regulation in comparison with the wild type, including 11 lncRNAs. One of them, now known as *ASCO*-RNA for Alternative Splicing Competitor RNA, was found to bind the genes by competition with the NSR proteins, inducing a change in the gene-splicing pattern and altering the auxin-mediated lateral root development phenomena (Bardou et al., 2014).

#### 6.1.3 Photomorphogenesis

The plant’s response to light plays an important role in development. Photomorphogenesis is an important phenomenon modulated by light, which is responsible for the essential morphological changes during the vegetative and reproductive phases of plants, such as hypocotyl growth, pollen development, and phototropism, among others. Since early in the study of plant lncRNAs, they have been found to play crucial roles in these light-related responses. In *A. thaliana*, it was found the lncRNA Hidden Treasure 1 (*HID1*), which inhibits the transcription of the gene encoding the transcription factor PIF3, is a negative regulator of photomorphogenesis, related to the plant response to red light. *HID1* participates in the creation of protein-RNA, which interacts with the first intron sequence of the *PIF3* gene to inhibit its transcription in *cis*. Functional evidence for *HID1* was obtained by studying the *hid1* mutant, which shows a large expression of PIF3 protein and a hypocotyl growth response under red light conditions (Wang et al., 2014a).

In rice, an lncRNA of 1,236 nt controls the development of pollen grain during days with more hours of light. This lncRNA shows a large expression during long-day conditions, and it was designated as *LDMAR* for Long-Day Specific Male Sterility-associated RNA. Rice mutants for this transcript display an aberrant pollen grain development by the activation of the programmed cell death phenomena and a male sterile phenotype, consequently (Ding et al., 2012).

In *Zea mays*, an lncRNA with 269 nt in length, designated *zm401*, plays an important role in anther development. *Zm401* is lowly expressed during the formation of floret and has an increased expression in the mature pollen grain. By reducing the *zm401* expression to 10% with transgenic plants overexpressing the gene in sense orientation and RNA interference, it was shown that this lncRNA alters the expression of *MZm3-3*, *ZmMADS2*, and *ZmC5* genes, which plays different roles during anther development and stamen growth. Furthermore, orthologues of this lncRNA were identified in rice, wheat, and millet, suggesting that the pollen development phenomenon is highly conserved (Ma et al., 2008). Similarly, in *Brassica campestris*, an lncRNA of 828 nt designated as *BcMF11* participates in the control of normal pollen development. By inhibiting the expression of this lncRNA through antisense technology, it was found that the transgenic plants showed lower germination of the pollen grains and an arrested pollen tube development. This phenotype was due to an abnormal tapetum degradation and abnormal development of pollen grains (Song et al., 2013).

The responses to red and far-red light have also been studied in the plant *Dendrobium officinale* using RNA-seq from plants that were treated with different red, blue, and far-red light levels. Reads generated were mapped to the *D. officinale* reference genome (Zhang et al., 2016). A total of 3,770 lncRNAs were found, with seven upregulated and four downregulated in comparison to the control. The lncRNA–mRNA interaction network created showed that some of the lncRNAs can target up to 20 different genes based on the genome location, 10 kb or 100 kb upstream and downstream, respectively, from the lncRNA. It was found that the lncRNA can alter the responses of the plant to the red and far-red light by controlling the cell signal and perception of light, several metabolic pathways, and hormone signal transduction, and even by inducing epigenetic changes through the changes in the activity of methyltransferase enzymes (Li et al., 2021).

#### 6.1.4 Leaf development

In plants, the leaves have an essential role as they are responsible for gas exchange, thermoregulation, and photosynthesis, among others. Its development and physiological changes are subject to a complex network of transcriptional regulation, which is also mediated by ncRNAs such as lncRNAs. Based on experimental data, the lncRNA *TWISTED LEAF* (*TL*) was identified as a regulator of leaf development in *Oryza sativa*. *TL* lncRNA is antisense to a gene encoding the transcription factor OsMYB60. Overexpression of this transcription factor confirmed the same phenotype observed in the lncRNA deletion, which is a twisted leaf blade. In addition, the expression level of the *OsMYB60* gene showed a significant increase in plants that had the *TL* gene deleted, demonstrating that the *TL* lncRNA plays an important role in the regulation of leaf development in rice, probably as a *cis* regulator of the *OsMYB60* gene through chromatin modifications (Liu et al., 2018). In Arabidopsis, multiple lncRNAs associated with leaf development using high-throughput sequencing data. Indeed, 746 lncRNAs were found to be expressed in leaves, showing significant changes in their expression patterns in the late stages of leaf development. Of these lncRNAs, 28 are part of the complex regulatory network of leaf development mediated by the interaction between competitive endogenous RNA (ceRNA) and circular RNAs (circRNA) (Meng et al., 2018). In *Eucalyptus grandis*, the gene expression of leaf and the leaf stem tissues obtained from 5-month trees was compared using RNA-seq, leading to the identification of 551 lncRNAs. Of these, 130 and 124 were found exclusively in the leaf and stem, respectively. Furthermore, 297 were found to be present in both tissues. Both lncRNAs with target genes in *cis* and *trans* were identified. In *cis*, the most important phenomenon recorded was the ubiquitin-mediated proteolysis pathway. In this case, several interactions were recorded, namely, one lncRNA targeting one gene, several lncRNAs targeting one gene, and a single lncRNA targeting multiple genes. In the case of genes targeted in *trans*, it was found that the main pathways under control were protein export, protein processing in the endoplasmic reticulum, and phagosome. Moreover, a complex relation was found between lncRNA and miRNA to control gene expression. As an example, it was found that one miRNA aligned perfectly with one intergenic and leaf stem-specific lncRNA (Lin Z. et al., 2019).

#### 6.1.5 Fruit development and ripening

Fruits are a delicious treat for our taste and play a significant role in plant reproduction and propagation. The development and ripening of fruits are regulated by a coordinated and complex molecular mechanism that involve the modulation of expression levels of several genes and signaling networks. While protein-coding genes and miRNAs have long been recognized as key players in fruit development and ripening (Karlova et al., 2014; Fenn and Giovannoni, 2021; Arazi and Khedia, 2022), recent studies have shed light on the crucial role of lncRNAs in these phenomena.

In tomatoes, a study searched for lncRNAs within 134 RNA-seq sequencing data sets from 18 different tissues, including fruit, root, cotyledons, and flowers, among others (Wang et al., 2018). In tomato fruit, 14 data sets were generated from tomatoes in mature green, breaker, and breaker plus 7 days of stages of fruit development. This study revealed 70,635 lincRNAs, 8,085 antisense lncRNAs, and 602 sense lncRNAs. As a criterion, lncRNAs with fragments per kilobase per million (FPKM) values of more than 10 were considered to play a role in fruit ripening (Wang et al., 2018). Following this, 4,079 were found in fruit with mature green stage, 4,135 in fruit at breaker stage, and 4,311 in the fruit with breaker stage plus 7 days. Also, it was reported that 108 lincRNAs were differentially expressed in the mature green and breaker, 191 in mature green and breaker plus 7, and 16 in breaker and breaker plus 7. None of these lncRNAs were functionally characterized in this study; however, the information generated strongly suggests that they play an important role in the fruit ripening phenomena (Wang et al., 2018). On the other hand, based on RNA sequencing, Ou et al. (2017) identified 2,505 lncRNAs that may be related to hot pepper (*Capsicum annuum* L.) fruit development. Of these, 1,066 lncRNAs were associated with *cis*- or *trans*-acting gene targets involved in hormone signal transduction and cell wall formation, among others. This study suggested that lncRNAs are important players in hot pepper fruit development. In addition, Zhu et al. (2015) reported that 3,679 lncRNAs were identified by studying ripening mutant tomato fruit. The comparison with wild-type tomatoes showed 677 lncRNAs differentially expressed, suggesting an important role of these lncRNAs in fruit ripening. The silencing of two lncRNAs (*lncRNA1459* and *lncRNA1840*) induced a delay in ripening, demonstrating their participation as regulators of tomato fruit ripening.

A study using RNA-seq was performed in three developmental stages of the sea buckthorn fruit, *Hyppophae rhamnoides* L., which corresponds with the mature green, breaker, and red ripe stages of development. The authors recorded 9,008 lncRNAs, and out of these, only 13.4% and 11.7% were found to be intronic lncRNA and natural antisense lncRNA, respectively. Using as a criterion the presence of genes 100 kb upstream and downstream, it was found that the differentially expressed lncRNA control in *cis* different physiological phenomena in the fruit. Furthermore, it was recorded that 22 lncRNAs can be playing a role as plant endogenous target mimics for 25 differentially expressed miRNAs. In this regard, using a virus-induced gene silencing approach, it was shown that two lncRNAs acting as plant endogenous target mimics for the *miR156a* and *miR828a* can change the expression of the transcription factors *SPL9* and *MYB114*, inducing an increase or decrease in the concentration of fruit anthocyanins, depending on the lncRNA repressed (Zhang L. et al., 2018).

In addition, based on RNA-seq analysis in strawberry (*Fragaria vesca*) fruits, it was possible to identify 5,884 lncRNAs as possible regulators in flower and fruit development (Kang and Liu, 2015). Moreover, a genome-wide analysis in peach (*Prunus persica*) identified approximately 575 lncRNAs putatively related to fruit development and the ripening process (Zhou et al., 2022). Also, co-expression network analysis in apple (*Malus domestica*) reported lncRNAs associated with fruit ripening (Wang et al., 2022). To date, many lncRNAs have been identified using genomics approaches with probable roles in fruit development and the ripening process; however, their biological function is still not elucidated. Therefore, there is still much work to be done to better elucidate the physiological function of lncRNA in fruit development and ripening.

It is normal for plants to be exposed to various types of biotic and abiotic stresses. Because of that, they have developed diverse strategies involving structural changes, many genes, and regulatory networks to adapt and survive. Recently, evidence has been generated of the participation of an important number of lncRNAs in the plant response to stress. In the following sections, we will describe the main advances in this area.

## 7 Biotic stress

In Arabidopsis, the lncRNA designated as *ELENA1*, which stands for ELF18-induced lncRNA, was found by analyzing seedlings treated with elf18 (which comes from the N terminus of the protein translation elongation factor Tu). Analysis of transgenic plants created by eliminating or overexpressing the gene *ELENA1* showed that this lncRNA plays a role in response to the attack of *Pseudomonas syringae* pv tomato. Indeed, the elimination of the gene rendered the plants more susceptible, whereas plants overexpressing it showed more resistance. Furthermore, it was found that *ELENA1* was induced by plant treatment with either elf18 or flagellin. RNA-seq analysis was performed in the wild type and one of the lines overexpressed the *ELENA1* gene after elf18 treatment. A total of 535 and 603 protein-coding genes were upregulated in the wild-type and overexpressing lines, respectively. Analysis of the genes showed an enrichment in biological processes related to defense and immune responses, clearly showing that this lncRNA plays a role in activating the plant defense system (Seo et al., 2017). Furthermore, in *A. thaliana*, 15 antisense lncRNAs and 20 intergenic lncRNAs induced in response to *Fusarium oxysporum* infection were located. In the case of the intergenic, it was found that the elimination of five of them by RNA interference approach or T-DNA insertion induces faster development and more severe *F. oxysporum* infection symptoms. In the case of the antisense lncRNA, 10 and 5 were induced or repressed in tissues after 6 days of inoculation, respectively. Also, in two of them, the presence of a fungi attack response box in the promoter region was found. Furthermore, it was demonstrated that the induction of one gene and that of its corresponding antisense lncRNA were independent events. However, the elimination of the expression of one of the antisense lncRNA did not change the infection phenotype between RNA interference lines and wild type (Zhu et al., 2014).

In *Medicago truncatula*, the lncRNA *Enod40* with a size of 700 nt was found, designated as MtRBP1 for *M. truncatula* RNA binding protein 1. It was shown to interact with the protein RBP1 within the nucleus to induce the cytoplasm export of this protein during the plant cell differentiation before and after *Rhizobium meliloti* infection to start the symbiosis. It was found that this lncRNA can encode two small peptides, but it was demonstrated that the elimination of the ATG translation initiation codon in *Enod40* does not impair the interaction with the RBP1 (Campalans et al., 2004). Furthermore, the elimination of the region between the two small peptides does not alter the small peptide translation but impairs the biological activity of the *Enod40* lncRNA (Sousa et al., 2001).

Two nearly isogenic wheat lines were inoculated with *Puccinia triticina*, the causal agent of leaf rust. Sequencing by RNA-seq was carried out in leaves at 0 and 96 h post-inoculation. A total of 1,178 lncRNAs were found, 22 of which were differentially expressed, and 49 played a putative role as an endogenous target mimic for 76 miRNAs. Interestingly, one of the lncRNAs found appears to play a role in the biosynthesis of two miRNAs. The authors suggested that this information will provide a better understanding of the wheat response mechanism to fungi attack (Jain et al., 2020).

The role of lncRNAs during the infection of *Zizania latifolia* by *Ustilago esculenta* was studied. This work also studied the role of lncRNA in response to different temperatures during infection, but in this description, we will focus on the infection phenomena. It was found that during infection at 25°C, 144 and 106 lncRNAs were exclusively expressed in *Z. latifolia* and *U. esculenta*, respectively. Out of these, 91 genes in *cis* were identified as targets for the lncRNA differentially expressed in *Z. latifolia* and 4 genes in *U. esculenta*. In the case of *Z. latifolia*, transcriptions factors, cell wall genes, ATPases, photosynthesis complexes, and proteins of the mitochondria were found. Furthermore, it was shown that the bark storage protein is targeted in *cis* by one lncRNA. It was shown that the plant defense response is suppressed in *Z. latifolia* by lncRNA, which allows the symbiotic formation of the culm galls (Wang et al., 2021).

With the goal to study the role of miRNAs as well as lncRNA in the response of tomatoes to the infection by *Phytophthora infestans*, transgenic plants overexpressing the miRNA *slMIR482e-5p* under the control of the promotor CaMV35S were created. Three transgenic lines showed a higher level of disease development when infected with *P. infestans* compared to the isogenic lines. Also, a significant expression of the fungi actin gene was recorded, as well as a low expression level of the pathogenesis-related proteins SlPR1 and SlPR5 by RT-PCR in the transgenic lines. However, after tomato infection, a decrease in the expression of the *slmiR48232-5p* was found. Furthermore, an endogenous target mimic sequence in the lncRNA *sllncRNA39298* was found. The role of this lncRNA was shown by creating transgenic plants overexpressing the *sllncRNA39298*. In these transgenic plants, a very low expression level of the *slmiR482e-5p* was observed, as well as a lower degree of fungi infection symptoms, compared with the isogenic lines. Moreover, a low expression of the fungi actin gene and a large expression of the pathogenesis-related proteins SlPR1 and SlPR5 were recorded. Altogether, these data clearly show that the *sllncRNA39298* enhances the resistance of the tomato plant to *P. infestans* infection by inhibiting the expression of the miRNA *slmiR482e-5p* (Liu et al., 2022).

The role of lncRNA in the response of rice to the infection of *Magnaporthe oryzae* was studied. Rice was infected with *M. oryzae*, and RNA of infected and control plants was isolated at three time points after infection. Overall, 4,787 lncRNAs were found; out of these, 2,366, 2,184, and 237 were intergenic, NATs, and intronic, respectively. Furthermore, 161 differentially expressed lncRNAs were recorded between infected and control plants, and about half were found to be either up- or downregulated. A correlation analysis with 203 differentially expressed protein-coding genes and 35 differentially expressed lncRNA found that lncRNAs were expressed concurrently with genes related to defense response, terpenoid biosynthesis, jasmonate signal transduction, and transcription factors. Also, it was found that the intronic lncRNA *TU40741* is transcriptionally synthesized in opposite direction from the second intron of the gene LOX-RLL. This gene plays a role in jasmonic acid biosynthesis. Altogether, these data suggest that the lncRNA *TU40741* may play a role in the pathogen defense mechanism of rice (Wang et al., 2020).

Root samples were sequenced in two species of cotton with different resistances to *Verticillium dahlia*, which infects roots. *Gossypium barbadense* is resistant, and *Gossypium hirsutum* is susceptible. Six transcriptomes were generated from each species, and the lncRNAs identified showed large numbers of lincRNAs and lncNATs. Two lncRNAs, *GhlncNAT-ANX2* and *GhlncNAT-RlP7*, were shown to be upregulated a few hours post-inoculation. Moreover, these lncRNAs inhibit the expression of their paired genes *GhANX2* and *GhRLP7*, respectively. Furthermore, plants with the lncRNAs silenced showed higher resistance to the *Verticillium dahlia* fungi, suggesting that the lncRNAs carry out a negative effect on gene expression and, consequently, in the phenotype of fungi resistance. Also, an increase in the expression of *LOX1* and *LOX2* genes was found, which regulates the plant’s resistance to pathogens. Interestingly, the two silenced plants also showed better resistance to the infection by the fungi *Botrytis cinerea* (Zhang L. et al., 2018a).

## 8 Abiotic stress

To study the function of the lncRNA in the molecular responses to the heat of radish, *Raphanus sativus* L., the RNA of young leaves treated for 6 h at 40°C was sequenced. The bioinformatic analysis found differentially expressed mRNAs, lncRNAs, miRNAs, and circRNAs. Focusing on lncRNAs, 117 were found upregulated and 52 were found downregulated by the treatment. Furthermore, 47 and 25 were only found in treated and control tissues, respectively, while 2,584 lncRNAs were found in both tissues. The lncRNA function was predicted based on the analysis of simultaneous expression and location with the mRNA protein-coding genes. From these, it is worth mentioning that the mRNA of a heat shock protein was found to be similarly upregulated by an upregulated lncRNA. Considering the metabolic pathways, the highest number of lncRNA was found in the ribosome, the processing of proteins in the endoplasmic reticulum, carbon metabolism, and biosynthesis of amino acids. Also, by analyzing the KEGG pathways, the photosynthesis carbon fixation, oxidative phosphorylation, peroxisome, and hormone signal transduction were identified as enriched. Thus, the pathways of energy generation and synthesis of carbon play an important role in the heat response of radish and may be regulated by lncRNAs and or transcription factors upregulated in response to heat (Yang et al., 2019).

A study of the function of lncRNAs in response to the heat of Chinese cabbage (*Brassica rapa* L. ssp. *Pekinensis*) was carried out by analyzing the data stored in the National Agricultural Biotechnology Center, Republic of Korea (Zhang et al., 2018). The analysis showed the presence of 278 lncRNAs, which were classified into six categories instead of the common four in which they usually are classified. Furthermore, among the lncRNAs, lincRNAs were the most prevalent with 234, classified between unknown intergenic lncRNA and fragments generated by the transcriptional noise of the polymerase, which is typically located close to regions transcriptionally active. Genes located 100 kb upstream and downstream of the lncRNA location were considered to be under *cis*-regulation. Based on this analysis, 33 genes were predicted to be regulated in *cis* by the lncRNA. Furthermore, they were found to belong to five ontology terms. It was suggested that the genes encoding for the HSP40 and REF4-related 1, which stand for the mediator of RNA polymerase II transcription subunit 33 A, were controlled by the lncRNA 094 and 185, respectively (Eom et al., 2021).

To study the regulation of the response to cold by the plant *M. truncatula*, the leaves and roots of the plant were cold-treated and analyzed by RNA-seq. A total of 1,288 and 983 lncRNAs were found to be upregulated by the treatment in roots and leaves, respectively. With the goal to study the control of genes by lncRNAs, it was decided to study the regulation of the *CBF* genes, for C-repeat/DRE binding proteins. These genes are known to be part of the QTL on chromosome six, conferring cold tolerance to *M. truncatula*. Seven *CBF* genes and the lncRNA designated *MtCIR_1_* for *M. truncatula*-CBF-intergenic RNA are located on chromosome six. *MtCIR_1_* is an intergenic lncRNA located between two *CBF* genes, without overlapping. It was found that *MtCIR_1_* showed upregulation after 2 h, whereas the *CBF* genes followed the same behavior after 5 h. Furthermore, three of the *CBF* genes remained with a large expression level after 24 h. Based on these experimental lines of evidence, it was suggested by the authors that *MtCIR_1_* is related to the expression of *CBF* genes (Zhao et al., 2020).

The duckweed *Spirodela polyrhiza* was treated with 100 mM of NaCl to study the lncRNAs active in response to saline stress. This treatment reduced the relative growth rate of the species by approximately 60% after 96 h of treatment. RNA samples were taken at 0, 6, 12, and 24 h after treatment initiation. A total of 2,185 lncRNAs were identified, and out of these, 566 and 2,269 were shown to be antisense and intergenic, respectively. A total of 185 lncRNA were shown to be differentially expressed. In addition, 38, 32, and 25 lncRNAs were expressed exclusively after 6, 12, and 24 h. In brief, it was found that the differentially expressed lncRNAs carry out a transacting control of genes playing a role in photosynthesis, cell wall metabolism, and RNA transcription, among others. Furthermore, some of these lncRNAs were found to control genes in *cis*, playing a role in the cell wall, reactive oxygen species regulation, and transcription factors, among others. In addition, an interactive network of miRNAs and lncRNAs was constructed, allowing us to find lncRNAs targeted by microRNAs. The most important was the miRNA156, which is known to participate in different abiotic stresses and has been shown to target 40 lncRNAs. Based on the results mentioned, lncRNAs play an important role in the duckweed response to saline stress (Fu et al., 2020).

To study the function of lncRNAs in the rice response to saline stress, an experiment was carried out with a tolerant genotype (FL478) and a sensitive genotype (IR29). Specifically, 21-day-old seedlings were collocated in a Yoshida solution containing 150 mM of NaCl for 24 h, followed by RNA extraction and sequencing. A total of 15,131 and 16,256 sequences belonging to lincRNAs, intronic lncRNAs, antisense lncRNA, and sense lncRNA types were found in the FL478 and IR29 genotypes, respectively. Correspondingly, four and nine differentially expressed lncRNAs were identified in FL478 and IR29. The most important of them is the *LncRNA.2-FL* of the FL478 resistant genotype, which appears to control 172 mRNA in *trans* and a gene encoding a pentatricopeptide repeat in *cis*. In summary, the function of the *lncRNA.2-FL* is to induce the lateral root development by the redirection of auxin with the goal to avoid the large concentration of salt (Mansuri et al., 2022).

The response to cadmium in rice roots in the early stages of development was studied by treating 2-day-old roots with 100 mg L^−1^ cadmium solution for 5 days. A clear inhibition of root development was found in the treated rice seedlings. The gene encoding lncRNA was confirmed by choosing the lncRNA found in common by the search in the protein family database and the CPC software. Out of the 144 lncRNAs identified and found to be differentially expressed, 120 were intergenic lncRNAs, 23 were antisense lncRNAs, and 1 was intronic lncRNA. The genes under *cis* control of the lncRNA were analyzed by looking 10 kb upstream and 100 kb downstream of the lncRNA genome location. Furthermore, genes under *trans* control were localized by studying the changes in expression level. In total, genes playing a role in 17 pathways were shown to be under lncRNA *cis* control. These pathways included photosynthesis, amino acids, carotenoids, sulfur metabolism, and secondary metabolism. On the other hand, genes under lncRNA control in *trans* were shown to play a role in 118 different pathways, including photosynthesis, secondary metabolites, biosynthesis of phenylpropanoids, and phenylalanine metabolism. These data show the importance of photosynthesis, secondary metabolism, and amino acid metabolism in the rice root response to the stress by cadmium (Chen et al., 2018).

To study the function of lncRNA in tobacco under low potassium concentration, 25-day-old tobacco seedlings were subjected to a treatment with 0.01 mM of potassium. The control treatment was tobacco seedlings with 2 mM of potassium. It was found that increased activity of the peroxidase and ascorbate-peroxidase enzymes in the seedlings under low concentrations of potassium indicated that the seedlings were under stress. Total RNA for sequencing was extracted from the roots and the shoots. A total of 11,742 lncRNAs were found; out of these, 8,853 were found to be lincRNA. Also, several lncRNAs were classified into 11 categories based on their position in the genome. Furthermore, 193 and 57 differentially expressed lncRNAs were identified in the roots and shoots of tobacco seedlings, respectively. Also, eight lncRNAs were found in both shoot and root tissues. Analysis of the constructed co-expression network found the interaction between 11 lncRNAs and three transcription factors. This suggests that the function of the lncRNA in low potassium stress takes place by transcription factor expression. By Gene Ontology analysis, it was shown that some of the enriched terms were a response to chemicals in roots, responses to an abiotic stimulus in roots and shoots, and response to oxygen levels in roots and shoots, among others. Based on the above data, the tobacco response to potassium starvation is complex and involves both roots and shoot tissues (Chen et al., 2022).

The role of lncRNA in the response of chickpeas to saline stress was studied by RNA-seq data of plant roots of two resistant and two susceptible varieties treated with 150 mM of NaCl. Genes under the *cis*-acting control of lncRNA were located by searching 10 kb upstream and downstream of the lncRNA genome location. Each salt-resistant line was compared with the two salt-susceptible lines to find the lncRNA role in salt stress response. Between 22–28 and 31–47 upregulated genes were found when comparing resistant lines with susceptible lines. Furthermore, the differentially expressed lncRNAs were shown to control in *cis* genes related to the response to saline stress like transporters, aquaporins, and transcription factors. Also, sequences corresponding to microsatellites were located in several lncRNAs. In addition, 80 lncRNAs were shown to be endogenous target mimics for 135 miRNAs. These data clearly suggest that the lncRNA can regulate the response to salt by controlling gene expression by acting in *cis* or as an endogenous target mimic (Kumar et al., 2021).

## 9 Conclusions and perspectives

The study of the genome non-coding elements is a challenging task, and this is especially true in the case of lncRNAs. This is mainly because they are transcribed from the exact genome locations of protein-coding genes, lack of sequence conservation, and show many similarities with the structure of genes. A promising approach for studying the lncRNAs is the analysis and inference of their secondary and tertiary structures; however, these characteristics are well known only for a few lncRNAs. Nonetheless, with the current level of development of next-generation sequencing technology and bioinformatic tools, the databases of non-coding genetic elements of different species will increase, making the identification and study of these important elements easier. Indeed, as of today there are several databases including PlncRNADB (Bai et al., 2019), EVLncRNAs (Zhou et al., 2021), Green Non-Coding (Di Marsico et al., 2022), PLNlncRbase (Xuan et al., 2015), LncPheDB (Lou et al., 2022), CANTATAdb 2.0 (Szczesniak et al., 2019), NONCODEV5 (Fang et al., 2018), LncReg (Zhou et al., 2015), PlantNATsDB (Chen et al., 2012), and JustRNA (Liao et al., 2018; Tseng et al., 2023). Currently, the studies available have shown the mechanism underlying the function that several lncRNAs play in different biological phenomena of plants, suggesting that these genetic elements represent an adjustable and versatile regulatory mechanism of the genome. It is clear that the study of lncRNAs in plants is still in its infancy, and much remains to be uncovered. Further research is needed to unravel the specific mechanisms by which lncRNAs regulate fruit and plant development processes. We expect that new lncRNAs will be found in the future, and more mechanisms of gene regulation by lncRNAs will be elucidated in plants.

As detailed above, lncRNAs have emerged as essential players in the orchestration of plant development. Their ability to regulate gene expression, modulate epigenetic modifications, and interact with other regulatory molecules highlights their significance in plant biology. Understanding the roles of lncRNAs in plant biology holds promise not only for gaining fundamental insights into the complex regulatory networks governing their development, growth, and stress response but also for the potential application in the improvement of different plant characteristics such as yield, protein content, reduction in the concentration of toxic compounds, fruit postharvest shelf life, amount of different nutrients and compounds with positive effects on human health, and the resistance to biotic and abiotic stresses.

## Author contributions

ED-R: Writing – original draft. MÁH-O: Writing – review & editing. SF-V: Writing – review & editing. MET-H: Conceptualization, Writing – review & editing.
